# A High Accuracy Pedestrian Detection System Combining a Cascade AdaBoost Detector and Random Vector Functional-Link Net

**DOI:** 10.1155/2014/105089

**Published:** 2014-05-19

**Authors:** Zhihui Wang, Sook Yoon, Shan Juan Xie, Yu Lu, Dong Sun Park

**Affiliations:** ^1^Department of Electronics Engineering, Chonbuk National University, Jeonju 561-756, Republic of Korea; ^2^Department of Multimedia, Mokpo National University, Jeonnam 534-729, Republic of Korea; ^3^Institute of Remote Sensing and Earth Science, Hangzhou Normal University, Hangzhou 311121, China; ^4^IT Convergence Research Center, Chonbuk National University, Jeonju 561-756, Republic of Korea

## Abstract

In pedestrian detection methods, their high accuracy detection rates are always obtained at the cost of a large amount of false pedestrians. In order to overcome this problem, the authors propose an accurate pedestrian detection system based on two machine learning methods: cascade AdaBoost detector and random vector functional-link net. During the offline training phase, the parameters of a cascade AdaBoost detector and random vector functional-link net are trained by standard dataset. These candidates, extracted by the strategy of a multiscale sliding window, are normalized to be standard scale and verified by the cascade AdaBoost detector and random vector functional-link net on the online phase. Only those candidates with high confidence can pass the validation. The proposed system is more accurate than other single machine learning algorithms with fewer false pedestrians, which has been confirmed in simulation experiment on four datasets.

## 1. Introduction

Nowadays, pedestrian detection has drawn the attention of many researchers, due to its wide range of applications, such as driver assistant system [1–3], intelligent video surveillance system [4, 5], and victim rescue in case of emergency [6]. Numerous pedestrian detection algorithms have been proposed during the past decades, based on different techniques and strategies [7–10].

Pedestrians have properties of both rigid and flexible objects. Furthermore, the appearances of pedestrians are easily affected by view angle, occlusion, apparel, scale, pose variation, and illumination changes. All these issues have made pedestrian detection become a hot issue and one of the difficulties in the fields of computer vision. In current mainstream methods for pedestrian detection, machine learning algorithms are adopted to distinguish and identify pedestrians from candidates extracted by multiscale sliding windows. However, high accuracy detection rates of these algorithms are always obtained at the cost of a large amount of false pedestrians. These experiments show that high accuracy detection rates and low false positive rates are by no means simultaneously guaranteed.

The two-stage classifier [11], proposed by Guo et al., can further reduce false positive rates and this system has better performance than these single-stage algorithms. However, the detection rates cannot be further increased and maintained at a certain level as can these single-stage algorithms. In this paper, a novel two-stage detecting system is proposed based on a cascade AdaBoost detector [9] and random vector functional-link net [12, 13]. These two algorithms can simultaneously deal with the normalized candidates extracted by multiscale sliding windows, which can guarantee the detecting efficiency of the proposed system. These processing results of the cascade AdaBoost detector and random vector functional-link net are fused together, as the final evaluation criteria of whether these candidates are pedestrians or not. The cascade AdaBoost detector and random vector functional-link net are two of the significant high-efficient machine learning algorithms. They have both been applied in many research fields, such as multimedia processing, natural language processing, biological information processing, and network security.

The proposed system can achieve high accuracy detection rates on the basis of low false positive rates, which is benefited from the joint promotion of the cascade AdaBoost detector and random vector functional-link net. The high performance of the proposed system has been demonstrated on four datasets, with different types, during our simulation experiments. The remainder of the paper is organized as follows. We start by introducing the structure of the proposed system in Section 2, and the experimental comparison of the proposed system with other state-of-the-art detectors is demonstrated in Section 3. Finally, we summarize the characteristics of the proposed system and discuss its superiority over other detectors in Section 4.

## 2. Proposed Pedestrian Detection System

As there is seldom any single detector that can reach excellent performance with high detection rate and few false positives in complex scenarios, the proposed pedestrian detection system is based on machine learning algorithms. The cascade AdaBoost (CAB) detector  [9] and random vector functional-link (RVFL) net  [12, 13] have been employed and combined to enhance the corresponding performance of detection results.

### 2.1. System Architecture

**Figure 1:**
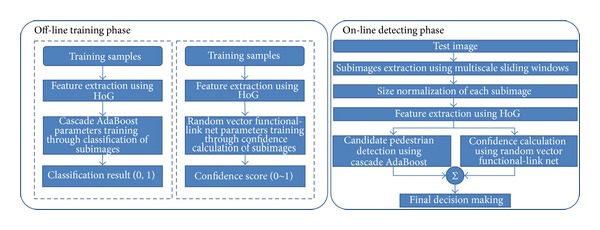
Flow chart of the proposed pedestrian detection system. The proposed pedestrian detection system contains an off-line training phase and an on-line detecting phase. These parameters of CAB detector and RVFL net are trained on the off-line training phase with training samples. All these testing subimages are extracted and verified to be targets or not during the on-line phase.

The flow chart of the proposed pedestrian detection system is demonstrated in Figure 1. The proposed system contains the off-line training phase and the on-line detecting phase. During the off-line training phase, the CAB detector and RVFL net are trained separately with the given training dataset. Each training sample has the same size, called the standard size, which is demonstrated in Section 3. The CAB detector is trained as classification pattern, while RVFL net is trained as regression pattern. For the classification pattern of CAB detector, the positive samples are labeled as 1 and negative samples are labeled as 0. During the training process of RVFL net with regression pattern, the confidence scale is limited in [0,1].

During the on-line detecting phase, all the subimages are generated by multiscale sliding windows, and they are resized to be the standard size as testing candidates. Then the CAB detector and RVFL net are employed to judge whether each candidate is a pedestrian or not. The CAB detector estimates whether each candidate is a pedestrian or not, and RVFL net estimates a confidence score for each candidate. Their two results are combined to get the final matching score and, finally, only those candidates with higher matching scores than the given threshold are regarded as pedestrians. The details of the proposed system are as follows.

### 2.2. Feature Extraction

Feature extraction is a type of dimensionality reduction that efficiently represents the ROI region of an image in the fields of object detection and pattern recognition algorithms. These features are extracted as a compact feature vector, for subsequent processing. Therefore, effective image feature extraction is rather important, which concerns final objection detection accuracy. Common features extraction techniques include the RGB histogram, local binary patterns (LBP) [14], histogram of oriented gradients (HOG)  [7], Haar-like feature, first-order image statistics (the mean standard deviation, skewness, and kurtosis of pixel intensities), second-order image statistics (the mean and range of contrast, correlation, energy, and homogeneity)  [15], and Hu's invariant matrix  [16].

Past research has shown that, in the past researches, Haar-like and LBP features have been used for detecting faces, as they have desirable properties for representing fine-scale textures. And the HOG features, which can capture the overall shape of an object, have been used for detecting objects such as people and cars. In this paper, HOG features are adopted to enhance the pedestrian detection performance of the proposed system. In our experiment, the parameters for the HOG feature extraction applied to the CAB detector and RVFL net are the same. For our system, the normalized candidates are divided into 16 × 16 pixel blocks; each block contains 2 × 2 cells of 8 × 8 pixels; linear gradient voting into 9 orientation bins in (0°, 180°). Therefore, the HOG features for CAB detector and RVFL net can be extracted in one step.

### 2.3. Cascade AdaBoost Detector (CAB)

**Figure 2:**
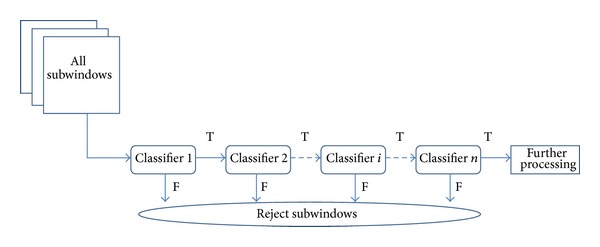
Flow chart of the cascade AdaBoost detector. Each classifier in cascade AdaBoost detector works independently, and the minimum true positive and the maximum false alarm rates of these stages are the same. Only these subwindows accepted as true positives by all stages of the detector are regarded as targets. “*T*” means that true candidates of these subwindows passed the verification of each classifier, and “*F*” means that these false candidates are rejected by the corresponding classifier.

The cascade AdaBoost algorithm [9] is adopted, to detect object categories whose aspect ratio does not significantly vary. This algorithm consists of a series of classifiers, where each classifier is an AdaBoost learner and its parameters are adjusted utilizing a boosting algorithm. The flow chart of the cascade AdaBoost algorithm is illustrated in Figure 2. The expression of the cascade AdaBoost algorithm is formed as

(1) $$\begin{array}{c} H\left(x\right)=\{\begin{array}{cc} 1, & H_{i}\left(x\right)=1,\;i=1,\ldots ,n;\\ 0, & \text{otherwise}, \end{array} \end{array}$$

where **x** is sample inputs, *n* is the number of stages, and *H*_*i*_ is the strong classifier of stage *i*, which can be represented as

(2) $$\begin{array}{c} H_{i}\left(x\right)=\{\begin{array}{cc} 1, & \overset{m}{\underset{j=1}{\sum }}\alpha_{ij}h_{ij}\left(x\right)\geq \frac{1}{2}\overset{m}{\underset{j=1}{\sum }}\alpha_{ij};\\ -1, & \text{otherwise}, \end{array} \end{array}$$

where *m* is the number of weak classifiers of each stage, *h*_*ij*_ is the *j*th weak classifier, and *α*_*ij*_ is the corresponding ensemble weight of *h*_*ij*_. Suppose the total number of positive samples is *N*, and the minimum true positive rate is *η*; then the number of positive samples to use at each stage is calculated by

(3) $$\begin{array}{c} N_{\text{stage}}=\left\lfloor \frac{N}{1+\left(n-1\right)\times \left(1-\eta \right)}\right\rfloor , \end{array}$$

where ⌊·⌋ is the floor function. The number of negative samples for each stage is always set to be 2*N*_stage_, twice the positive samples.

During the training process, a certain amount of positive samples and negative images are required. The feature type and number of stages are set and other function parameters, which contain the minimum true positive and the maximum false alarm rates, are first initialized. Then, the parameters of each stage are estimated with partial positive and negative samples.

As mentioned above, true positives are usually not sufficiently given and worth taking the time to verify through the cascade stages. Furthermore, sufficient negative samples should be provided to ensure the training phase is carried out smoothly, and typical negative samples are supplied containing background information of the images to be detected. During the parameter estimation of each stage, the AdaBoost learner is trained by adding features, until the minimum true positive and the maximum false alarm rates are met. The number of stages is determined with proper final false positive and detection rates.

During the detection phase, as shown in Figure 2, all subwindows of the image are extracted through a multiscale sliding window. The structure of the cascade AdaBoost reflects that the vast majority of these subwindows are negative. As such, each stage of the cascade AdaBoost detector rejects the large possible number of nonpedestrian windows and lets potential targets pass to the next stage. Finally, only a few of these subwindows accepted by all stages of the detector are regarded as objects.

### 2.4. Random Vector Functional-Link (RVFL) Net

The random vector functional-link net [12, 13] is a special case of the single hidden layer feed-forward neural network. The hidden layer contains two different types of nodes: input patterns and enhancement patterns. Input patterns are simple linear combinations of sample inputs. These additional enhancements can be represented as *g*(**a**_*j*_^*t*^ **x** + *b*_*j*_), where **a**_*j*_ is the weights of the input vector, *b*_*j*_ is the threshold parameter for the *j*th node, **x** = [*x*_1_,…, *x*_*N*_] is the sample inputs, and *g*(·) is the activation function. Therefore, the RVFL net can be interpreted as a mapping from *N*-dimensional space to (*J* + *N*)-dimensional space, where *N* is the dimensionality of training sample inputs, and separately, *J* is the number of additional enhancements. The output of the RVFL net can be represented as

(4) $$\begin{array}{c} f\left(x\right)=\overset{J}{\underset{j=1}{\sum }}\beta_{j}g\left(a_{j}^{t}x+b_{j}\right)+\overset{J+N}{\underset{j=J+1}{\sum }}\beta_{j}x_{j}. \end{array}$$

For the random vector functional-link net, **a**_*j*_ and *b*_*j*_ are randomly generated according to an appropriate given distribution (e.g., Gaussian distribution). Therefore, only the weight vector **B** = [*β*_1_, *β*_2_,…, *β*_*J*+*N*_] needs to be learned, which largely reduces the time cost of the training phase. The optimal weight vector **B** is obtained by minimization of the system error

(5) $$\begin{array}{c} B=\text{arg min}\left\{\frac{1}{2P}\overset{P}{\underset{p=1}{\sum }}{\left(t^{\left(p\right)}-Bd^{\left(p\right)}\right)}^{2}\right\}, \end{array}$$

where *P* is the number of training samples, **d** = [*g*(**a**_1_^*t*^ **x** + *b*_1_),…, *g*(**a**_*J*_^*t*^ **x** + *b*_*J*_), *x*_1_,…, *x*_*N*_] is the enhanced pattern vector, the subscript (*p*) is the sample index, and *t* ^(*p*)^ is the target value of the *p*th training sample.

The unique minimum of system error can be found by a learning phase, such as the conjugate gradient approach [17, 18]. If matrix inversion with the use of a pseudoinverse is feasible, then the optimal weight vector **B** is obtained by a single step, without any iteration. For this case, the pseudoinverse of optimal weight vector **B** was estimated by a single step in our experiments.

### 2.5. Matching Score Fusion

The proposed system deploys CAB and RVFL net to get more accurate detection rate. To obtain the final matching score for any subwindow, the proposed system fuses their two results: classification result *H*(**x**), represented by 0 or 1, from CAB and confidence score *f*(**x**), represented by continuous value with the range of (0,1), from RVFL net. Subimages with high matching scores can be accepted as objects. The function of matching score fusion is defined as

(6) $$\begin{array}{c} P_{\text{final}}\left(x\right)=f\left(x\right)+\lambda H\left(x\right). \end{array}$$

With proper activation function, the enhancement patterns of RVFL net are more powerful than these input patterns, as the output of enhancement patterns has nonlinear correlation with sample inputs. During our experiment, only enhancement patterns of RVFL net are employed, and the activation function is set to be a sigmoid function. For this case, the final match score *P*_final_(**x**) in (6) can be simplified to be

(7) $$\begin{array}{c} P_{\text{final}}\left(x\right)=\overset{J}{\underset{j=1}{\sum }}\beta_{j}g\left(a_{j}^{t}x+b_{j}\right)+\lambda H\left(x\right). \end{array}$$

## 3. Experiments

In this section, we compare our proposed two-stage detection system with four of the latest state-of-the-art detectors. To validate our proposed system, we have tested it on four publicly available sequences, which are PET'09 S3.MF (Multiple Flow) and PET'09 S0.CC (City Center) from PET benchmark [19], the “USC pedestrian set A” sequence from USC dataset [20], and the INRIA Person dataset  [21]. The first two datasets are consecutive frames captured by one fixed camera, while the sequences of the latter two datasets are chosen from different scenarios. For the city center sequence, the first 100 frames are selected for testing, as the amount of this sequence is quite large, while all sequences of the other three datasets are adopted, during this experiment. For the INRIA dataset, parts of these images are resized, to guarantee that these pedestrians have similar size to the training dataset, as the pedestrian size scale of this dataset varied greatly.

The training data are the same for all these four testing dataset, which are selected from the NITCA pedestrian dataset [22], with image size of 32 × 80 pixels. However, in order to improve the performance of the proposed system, 600 nonpedestrian images from the Daimler dataset [23] are added to the negative training dataset of the cascaded AdaBoost algorithm. The number of positive training samples is 500 for the cascade AdaBoost algorithm while the number of negative samples is twice that of the positives. For RVFL net, the amount of positive and negative samples is the same and is set to be 3000.

**Figure 3:**
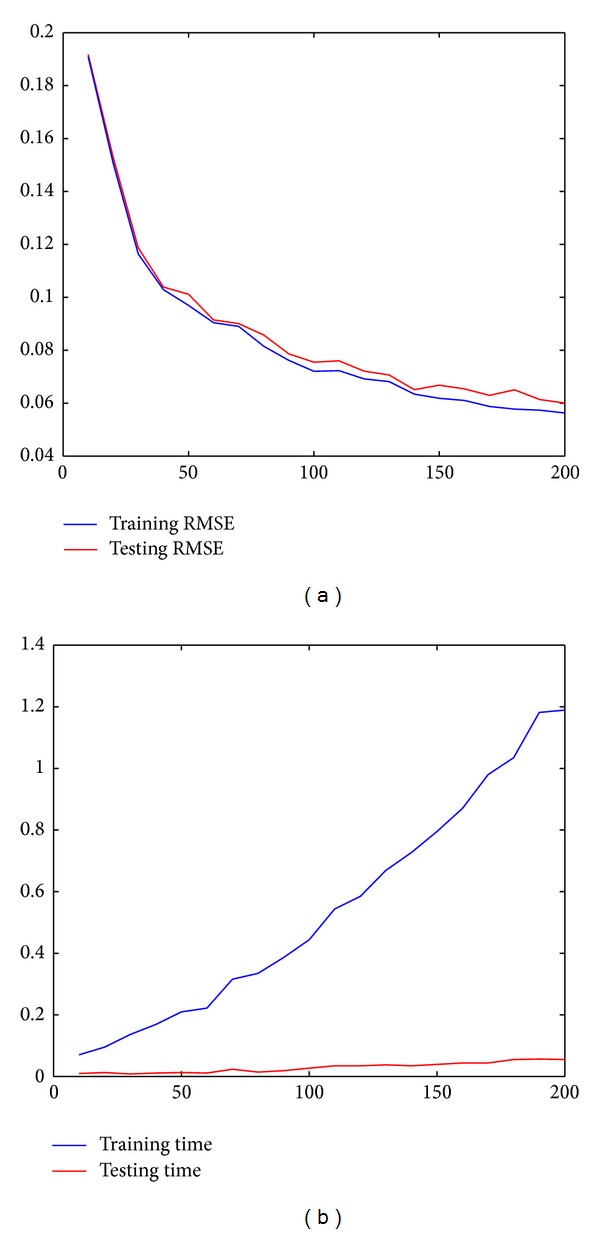
Performance comparison for RVFL net, with respect to hidden nodes. The *x*-axis of these two subimages represents the number of hidden nodes, which varied from 10 to 200 with step size 10. The left subimage is the training and testing RMSE and the right subimage is corresponding training and testing times.

Figure 3 shows the training and testing accuracies and times of RVFL net, with increasing number of hidden nodes from 10 to 200, by the step of 10. All these results are estimated by k-fold cross-validation [24]. During the cross-validation process, the whole dataset is randomly partitioned into 10 equal size subsets, and one single subset is selected as the validation data for testing the model, while the remaining 9 subsets are used as training data, on a case-by-case basis. Finally, the number of hidden nodes is set to be 180, with smooth and efficient training accuracy and high capability of generalization. When the number of hidden nodes is 180, the testing time is just 0.056 s, although the training time reaches 1.18 s. Therefore, the efficiency of RVFL net is guaranteed, during practical applications.

**Figure 4:**
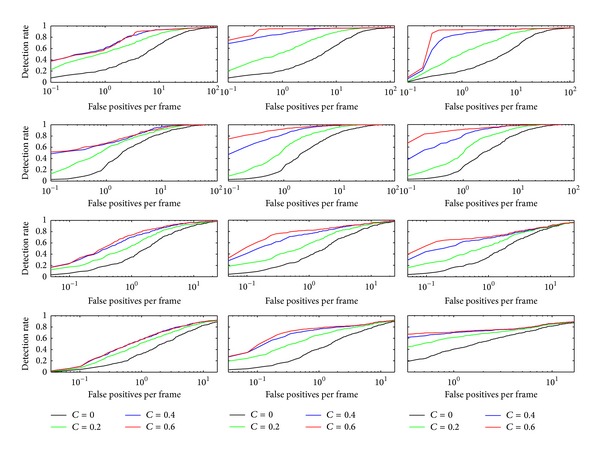
Comparison of parameter pair (*λ*, *n*). Each row of these subimages represents (detection rate/false positives per frame) curve for each dataset, which are “Multiple Flow,” “City Center,” USC(A), and INRIA dataset in order from top to bottom. The stage numbers *n* of these three columns are 8, 12, and 16 in order from left to right. Each subimage represents the performance of added confidence *λ* for given dataset with stage number *n*.

The minimum true positive and the maximum false alarm rates of CAB detector in our experiment are set to be 0.995 and 0.5, correspondingly.  Figure 4 shows the (detection rate/false positives per frame) curve of the proposed system with the parameter pair (added confidence *λ* and stage number *n* of the CAB detector). The formulas of the pedestrian detection rate (PDR) and false positives per frame (FPPF) are demonstrated as follows:

(8) $$\begin{array}{c} \text{PDR}=\frac{TP}{TP+FN}\times 100\%,\\ \text{FPPF}=\frac{FP}{N_{\text{frame}}}\times 100\%, \end{array}$$

where *TP* is the number of pedestrian samples correctly predicted to be pedestrians; *FP* is the number of nonpedestrian samples incorrectly predicted to be pedestrians; *FN* is the number of pedestrian samples incorrectly predicted to be nonpedestrians; *N*_frame_ is the number of total frames of the corresponding dataset sequences. We have tested the performance of added confidence *λ* with different values {0,0.2,0.4,0.6,0.8,1}. The curve of *λ* = 0.8,1 is very close to *λ* = 0.6, which means that the performance of the proposed system is beginning to stabilize when *λ* is growing larger than 0.6. The curve comparison of *λ* = 0,0.2,0.4,0.6 is demonstrated in Figure 4. From all these 12 subfigures, the performance of *λ* = 0.6 is superior to *λ* = 0,0.2,0.4. Moreover, when the stage number is 12, the results are better than *n* = 8,16, on the whole. Finally, the parameter pair is set to be (0.6, 12). Note that the single RVFL net can be regarded as a special case of the proposed system when *λ* = 0. Therefore, the proposed system has better performance than single RVFL net.

The comparison results of the proposed system and four other state-of-the-art detectors (CAB [9], SVM [7], GAB [25], and HF algorithm [8]) are shown in Table 1. In order to demonstrate the superiority of the proposed system, two sets of detection rates and the corresponding average number of false positives per frame of the proposed system are shown in the first two columns of Table 1. The second column shows high detection rates, at the cost of more false positives. However, its number of false positives per frame is still lower than SVM detector, in most cases. For “Multiple Flow” dataset, the low PDR of the proposed system is 94.55%, which is higher than those of the other four detectors. The corresponding FPPF, 0.56, is the lowest one among all these detectors. For “City Center” dataset, the low PDR and corresponding FPPF of the proposed system are 90.49% and 0.46, which are better than those for the GAB and HF algorithm. The high PDR reaches 95.29%, which is more accurate than CAB and SVM, at the cost of a few more false positives. For USC(A) dataset, the low PDR and corresponding FPPF are better than those for GAB and HF algorithm, and the high PDR and corresponding FPPF are better than those for SVM detector. The CAB algorithm has better performance of FPPF, while its detection rate is worse than the high PDR of the proposed system. For the INRIA dataset, the low PDR and corresponding FPPF of the proposed system are better than those for CAB, GAB, and HF algorithm, and the high PDR and corresponding FPPF are better than those for SVM detector.

**Figure 5:**
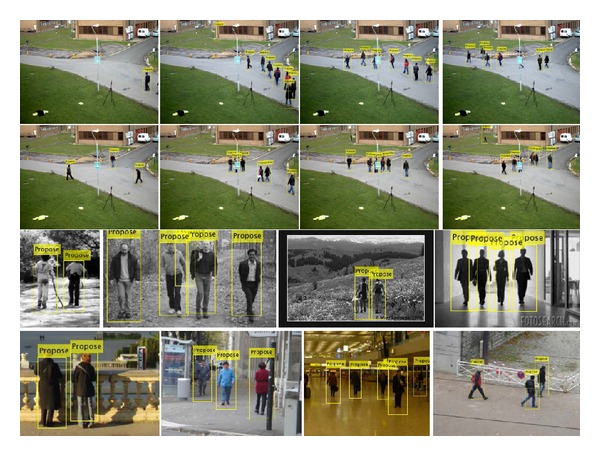
Examples of the four datasets. The first row is four samples of “Multiple Flow” dataset and the second row is “City Center” dataset. For demonstrated samples of these two datasets, each dataset has one pedestrian undetected due to the issue of heavy occlusion. The third row is USC(A) dataset and one false pedestrian is classified to be true positive target in the second subimage. The last row is INRIA dataset and all of the pedestrians are identified by the proposed system without any false pedestrians.

Parts of the experimental results of the proposed system are depicted in Figure 5. During the detection results of these examples, the overwhelming majority of these pedestrians are detected with very few false pedestrians, through the validation of the proposed system.

## 4. Conclusion

In this paper, we presented a novel two-stage pedestrian detecting system based on a cascade AdaBoost detector and random vector functional-link net. The proposed system simultaneously enhances the detection accuracy and reduces the false positive rate, which improves the comprehensive performance for pedestrian detection. Numerous experiment comparisons with other state-of-the-art algorithms on four challenging datasets with different types demonstrate that the proposed system achieves favorable results, in terms of the detection rate and false positive rate simultaneously.

## Figures and Tables

**Table 1:** Pedestrian detection rate (%) and false positives per frame comparison of CAB-ELM and other state-of-the-art detectors.

Data sets		Propose (low)	Propose (high)	CAB	SVM	GAB	HF
Multiple Flow	PDR	94.55	94.72	94.39	94.22	61.55	78.99
	FPPF	0.56	0.75	0.30	1.68	1.70	1.66

City Center	PDR	90.49	95.29	93.10	95.15	66.04	80.22
	FPPF	0.46	1.80	0.71	0.960	1.43	1.50

USC	PDR	73.16	81.79	81.15	80.83	36.74	34.82
	FPPF	0.24	0.88	0.34	0.91	0.34	0.42

INRIA	PDR	76.28	89.25	71.84	89.08	62.29	34.82
	FPPF	0.59	9.24	3.54	9.55	0.89	0.42

(1) The number of cascade stages of CAB and CAB-RVFL is 12.

(2) (low) and (high) means two sets with (low/high) detection rates and corresponding false positives per frame.

## References

[1] Gerónimo D, López AM, Sappa AD, Graf T (2010). Survey of pedestrian detection for advanced driver assistance systems. *IEEE Transactions on Pattern Analysis and Machine Intelligence*.

[2] Dollár P, Wojek C, Schiele B, Perona P (2012). Pedestrian detection: an evaluation of the state of the art. *IEEE Transactions on Pattern Analysis and Machine Intelligence*.

[3] Shashua A, Gdalyahu Y, Hayun G Pedestrian detection for driving assistance systems: single-frame classification and system level performance.

[4] Valera M, Velastin SA (2005). Intelligent distributed surveillance systems: a review. *IEE Proceedings—Vision, Image and Signal Processing*.

[5] Torresan H, Turgeon B, Ibarra-Castanedo C, Hebert P, Maldague X Advanced surveillance systems: combining video and thermal imagery for pedestrian detection.

[6] Andriluka M, Schnitzspan P, Meyer J Vision based victim detection from unmanned aerial vehicles.

[7] Dalal N, Triggs B Histograms of oriented gradients for human detection.

[8] Barinova O, Lempitsky V, Kholi P (2012). On detection of multiple object instances using hough transforms. *IEEE Transactions on Pattern Analysis and Machine Intelligence*.

[9] Viola P, Jones M Rapid object detection using a boosted cascade of simple features.

[10] Leibe B, Leonardis A, Schiele B (2008). Robust object detection with interleaved categorization and segmentation. *International Journal of Computer Vision*.

[11] Guo L, Ge P-S, Zhang M-H, Li L-H, Zhao Y-B (2012). Pedestrian detection for intelligent transportation systems combining AdaBoost algorithm and support vector machine. *Expert Systems with Applications*.

[12] Pao Y-H, Park G-H, Sobajic DJ (1994). Learning and generalization characteristics of the random vector Functional-link net. *Neurocomputing*.

[13] Park GH, Pao YH (2000). Unconstrained word-based approach for off-line script recognition using density-based random-vector functional-link net. *Neurocomputing*.

[14] Ahonen T, Hadid A, Pietikäinen M (2004). Face recognition with local binary patterns. *Proceedings of the 8th European Conference Computer Vision (ECCV '04)*.

[15] Theodoridis S, Koutroumbas K (2006). *Pattern Recognition*.

[16] Chen C-C (1993). Improved moment invariants for shape discrimination. *Pattern Recognition*.

[17] Fletcher R, Reeves CM (1964). Function minimization by conjugate gradients. *The Computer Journal*.

[18] Hestenes MR, Stiefel E (1952). Methods of conjugate gradients for solving linear systems. *Journal of Research of the National Bureau of Standards*.

[19] PETS 2009: Eleventh IEEE International Workshop on Performance Evaluation of Tracking and Surveillance. http://pets2009.net/.

[20] Wu B, Nevatia R Detection of multiple, partially occluded humans in a single image by bayesian combination of edgelet part detectors.

[21] INRIA dataset http://lear.inrialpes.fr/data/.

[22] Overett G, Petersson L, Brewer N, Andersson L, Pettersson N A new pedestrian dataset for supervised learning.

[23] Keller CG, Enzweiler M, Gavrila DM A new benchmark for stereo-based pedestrian detection.

[24] McLachlan GJ, Do KA, Ambroise C (2004). *Analyzing Microarray Gene Expressiondata*.

[25] Friedman J, Hastie T, Tibshirani R (2000). Additive logistic regression: a statistical view of boosting. *The Annals of Statistics*.

